# Advancing Whole-Person Health through Informatics: A Narrative Review of Knowledge Resources for Complementary and Integrative Health

**DOI:** 10.1016/j.tjnut.2026.101461

**Published:** 2026-03-02

**Authors:** Robin R Austin, Marcelo Fiszman, Evan Guerra, Rui Zhang, Halil Kilicoglu

**Affiliations:** 1 School of Nursing, Population Health Systems Cooperative, University of Minnesota, Minneapolis, MN, United States; 2 Semedy Inc, Newton, MA, United States; 3 School of Information Sciences, University of Illinois Urbana-Champaign, Champaign, IL, United States; 4 Division of Computational Health Sciences, University of Minnesota, Minneapolis, MN, United States; 5 Center for Learning Health System Sciences, University of Minnesota, Minneapolis, MN, United States; 6 Division of Nutritional Sciences, University of Illinois Urbana-Champaign, Urbana, IL, United States; 7 Personalized Nutrition Initiative, University of Illinois Urbana-Champaign, Urbana, IL, United States

**Keywords:** complementary therapies, whole-person health, complementary and integrative health, informatics, knowledge resources

## Abstract

**Background:**

Complementary and integrative health (CIH) interventions, including nutritional strategies, are widely used to support whole-person health, yet evidence on their efficacy, safety, and mechanisms remains fragmented.

**Objectives:**

This narrative review mapped existing CIH knowledge resources, identifies critical gaps, and highlighted challenges in interoperability and integration. We proposed artificial intelligence-driven informatics strategies to standardize, connect, and leverage these resources, with the goal of advancing discovery, precision nutrition, and personalized approaches to health and well-being.

**Methods:**

We conducted a narrative review of publicly available knowledge resources on complementary health interventions, focusing on their effectiveness, safety, and biological mechanisms, including the microbiome. Interventions were categorized as nutritional, physical, or psychological. Resources were then classified as knowledge bases, datasets, databases, ontologies, knowledge graphs, platforms, or initiatives, with summaries of their scope, functionality, and contributions.

**Results:**

We identified 47 resources that can support complementary and integrative health informatics (15 knowledge bases, 13 databases, 7 datasets, 4 platforms, 3 initiatives, 3 ontologies, and 2 knowledge graphs). Categories included nutritional interventions (32, with 13 on the microbiome), physical interventions (4), psychological interventions (3), and comprehensive or multimodal resources (7). Most resources (39) were publicly available.

**Conclusions:**

Advancing whole-person health requires greater standardization and integration of knowledge resources, which in turn enables more effective application of artificial intelligence and informatics methods. When well-structured, interoperable resources are coupled with these computational methods, they can unify diverse knowledge domains, advance the science of complementary and integrative health, and accelerate discovery in personalized nutrition.

## Introduction

Aging population, increase in chronic diseases, and soaring healthcare costs require a holistic, preventive, patient-focused approach to healthcare (*whole-person health*) [[1], [2], [3]]. Whole-person health refers to an approach that considers the entire individual across all domains of health, such as biological, behavioral, social, and environmental [2,3]. Complementary and integrative health (CIH) interventions, including natural products, nutritional interventions, and mind-body therapies, are widely adopted by healthcare consumers to support well-being and represent a critical frontier for precision nutrition and whole-person health. A nationwide health survey in 2012 showed that >30% of adults and 12% of children in the United States used some type of complementary intervention in the past 12 months, most commonly natural product supplements [4], with numbers increasing in subsequent years [5]. The scientific community and clinicians increasingly acknowledge the role of complementary interventions in human health [[6], [7], [8]], marking a shift from a disease-centric approach toward whole-person health.

**FIGURE 1:**
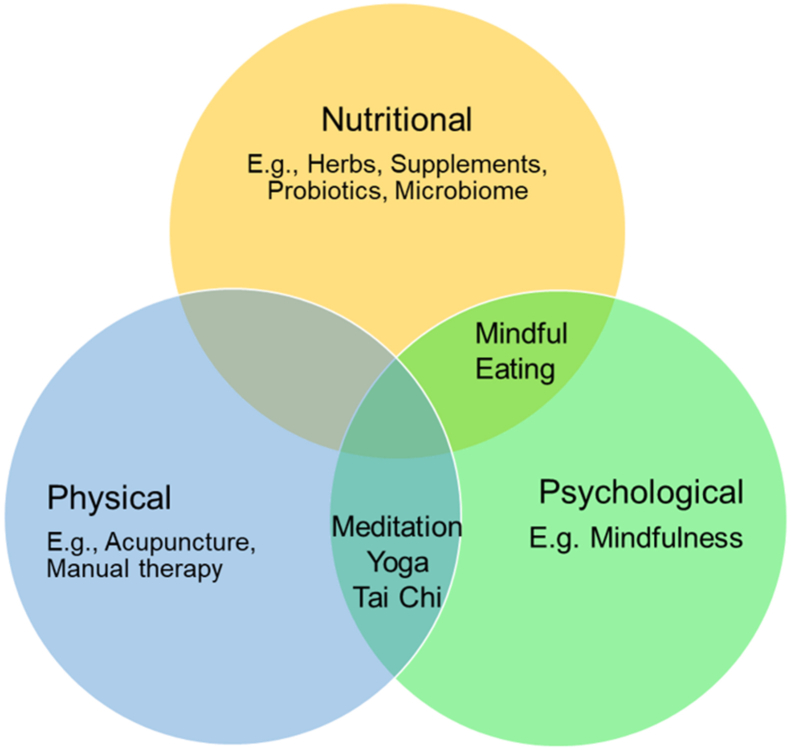
Simplified Complementary Health Categories based on The National Center for Complementary and Integrative Health (NCCIH) Strategic Plan 2021-2025.

CIH interventions typically encompass practices or systems originating outside conventional medicine. The National Center for Complementary and Integrative Health (NCCIH) organizes these therapies into 3 distinct but interconnected categories: *nutritional* (e.g., herbs, dietary supplements, probiotics, special diets, and microbial-based therapies), *physical* (e.g., acupuncture, massage, and spinal manipulations), and *psychological* (e.g., meditation, hypnosis, music therapies, and relaxation therapies) [9]. As our framework, we used NCCIH’s CIH classification, proposed in their Strategic Plan 2021-2025 (available at https://www.nccih.nih.gov/about/nccih-strategic-plan-2021-2025). A simplified version of the framework is shown Figure 1. This classification also acknowledges that effective CIH approaches often involve multicomponent interventions that simultaneously address nutritional (e.g., dietary recommendations), physical (e.g., exercise regimens), and psychological (e.g., mind-body stress reduction) factors, recognizing the inherent interconnections and combined influence on health outcomes.

Despite the growing popularity of CIH therapies, critical gaps remain in understanding their effectiveness, safety, and underlying biological mechanisms [10]. High-quality evidence on these topics remains limited [10], and current evidence is fragmented across multiple sources, hindering evidence-based practice. An integrative health approach requires accessible, structured resources that consolidate reliable information to support informed decision-making.

This narrative review examined current knowledge resources supporting CIH and their potential to advance precision nutrition and whole-person care. We identify critical gaps and highlight opportunities to apply artificial intelligence (AI)-driven informatics methods such as ontologies, natural language processing (NLP), machine learning, and knowledge graphs to enhance evidence synthesis, extract insights from unstructured data, and integrate diverse datasets, including clinical records, microbiome profiles, and multiomics measures. Leveraging these approaches can accelerate the generation of interoperable evidence on CIH therapies and enable more personalized, data-driven nutrition strategies.

## Methods

We conducted a narrative review to identify, describe, and synthesize key publicly available CIH resources. Between January and May 2025, a systematic search was conducted via Google Scholar and organizational websites. Using a snowball method, 2 authors (RRA and MF) identified CIH articles and resources by reviewing references and citation trails of key studies. Starting with a set of foundational or high-impact CIH articles, researchers expanded their search outward by following cited and citing works, enabling the discovery of additional relevant literature that may not appear through keyword-based database searches alone [11]. This method strengthens the depth and scope of CIH evidence reviews. The microbiome is a growing focus, recognized as a potential mediator of effects for complementary interventions (e.g., herbal medicine and acupuncture) [[12], [13], [14], [15]]. Therefore, this review places particular emphasis on knowledge resources related to microbiome. Keywords included: “integrative medicine,” “gut microbiome database,” “microbiome resources,” “microbial metabolite databases,” “diet-microbiome interactions,” “bioinformatics tools,” “exercise,” “sleep,” “yoga,” “mindfulness,” “meditation,” “tai chi,” “psychotherapy,” “social interactions,” “healthy lifestyle,” “acupuncture,” “dietary supplements,” “Chinese medicine,” and “medicinal plants,” and “probiotics.” Additional resources were identified through expert recommendations and reference mining. Resources were included if they: focused on CIH, human gut microbiome; provided genomic, metabolomic, dietary, clinical, or functional data; were publicly available for research use; and included tools for data analysis, exploration, or translational applications. All data were extracted and entered into an Excel spreadsheet. Data elements included resource name and organization and associated citations. We further categorized the identified resources using the NCCIH framework categories of nutritional, physical, and psychological, and synthesized to identify strengths, challenges, and future needs.

We also categorized the resources according to their type. Specifically, we distinguished datasets, databases, knowledge bases, ontologies, knowledge graphs, platforms, and initiatives, defined as follows:

• *Dataset:* a collection of raw or structured data, often organized in tables or files, and used for analysis, training machine learning models, or benchmarking.
• *Database:* a structured collection of data stored and managed electronically, with a query interface, with support for updates and indexing.
• *Knowledge base:* a curated collection of concepts, facts, and relationships, providing structured, high-quality knowledge for decision support and reasoning.
• *Knowledge graph*: a graph-based representation of entities (nodes) and their relationships (edges), representing and integrating heterogeneous knowledge, enabling inference and semantic querying.
• *Ontology:* a formal specification of entities in a domain, their properties, and the relationships between them, expressed in a logical language like OWL (Web Ontology Language).
• *Platform:* an integrated system or tool that provides access to various resources (datasets, tools, interfaces, and analytics) for a specific purpose, to enable users to interact with data or knowledge, perform analysis, or visualize results.
• *Initiative:* an organized effort, often collaborative and long-term, aimed at advancing a scientific goal, to foster research, resource development, policy, or community engagement.

This categorization helps clarify the goal of each resource and supports users in identifying the kinds of resources most relevant to their needs.

## Results

This review identified a wide range of resources (*n* = 47) specific to CIH interventions and the microbiome and included: knowledge bases (15); databases (13); datasets (7); platforms (4); initiatives (3); ontologies (3); knowledge graph (2) (Table 1) [[16], [17], [18], [19], [20], [21], [22], [23], [24], [25], [26], [27], [28], [29], [30], [31], [32], [33], [34], [35], [36], [37], [38], [39], [40], [41], [42], [43], [44], [45], [46], [47], [48], [49], [50], [51], [52], [53]]. Several freely accessible informatics resources link CIH interventions, such as food, herbal remedies, acupuncture-related concepts, and dietary supplements, to diseases, physiological processes, biological pathways, or gene targets. These databases and tools support network pharmacology and systems biology approaches by mapping multicomponent therapies (nutritional, physical, and psychological) to molecular mechanisms. Although these diverse resources offer valuable opportunities for CIH research and clinical application, they also present challenges due to variability in scope, data structure, and quality. We review and assess currently available resources, highlight their contributions, and identify gaps for future research and development.

**TABLE 1:** Knowledge resources examined in this review and their brief description

Resource name	Type	Access	Link	Citation	Description
Comprehensive					
Global Wellness Evidence	Platform	Public	https://globalwellnessinstitute.org/wellnessevidence/	—	Educational resource including expert-curated information from quality papers on preventive health and wellness. Developed by the Global Wellness Institute.
NatMedPro	Platform	Subscription-based	https://naturalmedicines.therapeuticresearch.com/	—	Authoritative resource on dietary supplements, natural medicines, and complementary, alternative, and integrative therapies.
NCCIH Health Topics (A–Z)	Knowledge base	Public	https://www.nccih.nih.gov/health/atoz	—	Authoritative evidence-based information on integrative medicine interventions to promote health.
NCI OCCAM	Knowledge base	Public	https://cam.cancer.gov/health_information/cam_therapies_a-z.htm	—	Authoritative evidence-based information on integrative medicine interventions for cancer.
VA Whole Health Library	Knowledge base	Public	https://www.va.gov/wholehealthlibrary/	—	Educational resource on complementary and integrative health strategies targeted for clinicians who work with veterans, but that are broadly relevant.
WHO Traditional and Integrative Medicine Mapping	Knowledge base	Public	https://www.who.int/health-topics/traditional-complementary-and-integrative-medicine#tab=tab_1	[16]	Large-scale effort commissioned by the WHO systematically mapping traditional, complementary, and integrative medicine interventions (TCIM) across all health conditions
Lifestyle Medicine Ontology (LSFO)	Ontology	Public	https://bioportal.bioontology.org/ontologies/LSFO	[17]	Structures lifestyle medicine concepts into a multihierarchical ontology.
Nutritional (incl. microbiome)					
Diet-Gut-Microbiome Resources					
curatedMetagenomicData	Dataset	Public	https://waldronlab.io/curatedMetagenomicData/	[18]	Provides standardized, curated human microbiome data for novel analyses (e.g., gene families, marker abundance).
Diet-Microbiome Relation Extraction (DiMB-RE)	Dataset	Public	https://github.com/ScienceNLP-Lab/DiMB-RE	[19]	Corpus of 165 nutrition and microbiome-related publications, developed for natural language processing.
Disbiome	Database	Public	https://disbiome.ugent.be/home	[20]	Database of microbial composition changes in different kinds of diseases, managed by Ghent University.
Food-Gut Microbiota Disease Interactions (FGMDI)	Dataset	Public (In manuscript)	No site	[21]	Comprehensive dataset for food-gut microbiota-disease interactions (FGMDI) analysis and dietary recommendation applications.
GMRepo	Database	Public	https://gmrepo.humangut.info/home	[22]	Curated database of consistently annotated human gut metagenomes.
Gut Microbiota Watch	Knowledge base	Public	https://www.gutmicrobiotawatch.org/	[23]	A website to provide reliable information on supplements and gut health.
Gut Microbial Metabolite Association with Disease (GMMAD V2.0)	Database	Public	http://gepa.org.cn/GMMAD2	[24]	A database that provides comprehensive metabolite-related information of gut microbes in human diseases.
gutMGene	Database	Public	https://bio-computing.hrbmu.edu.cn/gutmgene/#/home	[25]	Manually curated database to provide a comprehensive resource of target genes and microbial metabolites in humans and mice.
Human Gut Microbiome Atlas (HGMA)	Dataset	Public	https://www.microbiomeatlas.org/	[26]	Provides human microbiome data from human oral and gut samples obtained from several disease and healthy cohorts by integration of metagenomics and other omics data using systems biology.
Human Microbiome Compendium	Dataset	Public	https://microbiomap.org/	[26]	Dataset of human microbiome of over 160K samples of 16S rRNA amplicon sequencing data.
Human Microbiome Project (HMP)	Initiative	Public	https://commonfund.nih.gov/human-microbiome-project-hmp	[27]	A Common Fund project that developed research resources for the study of the microbial communities that play a role in human health and disease.
Microbial Metabolites Database (MiMeDB)	Database	Public	https://mimedb.org/	[28]	Freely available electronic database containing information about small molecule metabolites found in the human microbiome. Intended to be used for applications in metabolomics, clinical chemistry, biomarker discovery, and general education.
The Microsetta Initiative	Initiative	Public	https://microsetta.ucsd.edu/american-gut-project/	[29]	Informs citizen-scientist participants about their own microbiomes by providing a standard report and deposits all deidentified data into the public domain on an ongoing basis without access restrictions.
Virtual Metabolic Human (VMH)	Database	Public	https://www.vmh.life/	[30]	Captures information on human and gut microbial metabolism and links this information to hundreds of diseases and nutritional data.
Food, Dietary, and Herbal Resources					
Coconut	Platform	Public	https://coconut.naturalproducts.net/	[31]	Platform for natural product research; provides data, tools, and services for data deposit, curation, and reuse.
Diet-Drug Interactions Database (DDID)	Knowledge base	Public	https://bddg.hznu.edu.cn/ddid/	[32]	Manually curated, open-access knowledge base dedicated to diet-drug interactions research.
Encyclopedia of Traditional Chinese Medicine	Knowledge base	Public	http://www.tcmip.cn/ETCM/	[33]	Web-based TCM knowledge base established in 2018 for network analysis of TCM herbs and formulas.
FoodBall Portal	Initiative	Public	https://foodmetabolome.org/foodball	[34]	Multicentric project aiming to develop strategies for food biomarker discovery and validation, and to identify biomarkers of intake for a range of foods.
FoodData Central	Knowledge base	Public	https://fdc.nal.usda.gov/about-us	[35]	USDA’s comprehensive source of food composition data with multiple distinct data types.
FoodDB	Database	Public	https://foodb.ca/	[36]	Information about both macronutrients and micronutrients, including many of the constituents that give foods their flavor, color, taste, texture, and aroma.
FoodOn (LanguaL)	Ontology	Public	https://foodon.org/	[37,38]	Provides a comprehensive ontology built to interoperate with the international OBO Library collaboration and to represent entities that bear a “food role.” FoodOn is based largely on LanguaL.
High-throughput Experiment- and Reference-Guided Database (HERB)	Database	Public	http://herb.ac.cn/	[39]	An experiment and reference-guided database of traditional Chinese medicine.
Indian Medicinal Plants, Phytochemistry And Therapeutics Database (IMPPAT)	Database	Public	https://cb.imsc.res.in/imppat/home	[40]	Manually curated database which has been constructed via digitization of information from >100 books on traditional Indian medicine, 7000+ published research articles, and other existing resources.
Integrated Dietary Supplements Knowledge Base (iDISK)	Knowledge base	Public	https://conservancy.umn.edu/items/7dec9015-c428-44d3-80c7-625e0800d008	[41]	Covers a variety of dietary supplements, including vitamins, herbs, minerals, etc., their attributes and relationships.
NaPDI (Natural Product Drug Interactions)	Knowledge base	Public	https://napdicenter.org/	[42]	Provides access to scientific results, raw data, and recommended approaches to assess the significance of pharmacokinetic natural product-drug interactions.
NIH Office of Dietary Supplements	Platform	Public	https://ods.od.nih.gov/	—	Provides evidence-based summaries for health professionals and consumers on specific vitamins, minerals, herbs, and other dietary supplements.
Probiotics Reference Guide	Knowledge base	Public	https://dhd.digihealthdojo.com/probioticdb/	[43]	Informational resource designed to provide healthcare professionals with evidence-based information related to probiotic products to assist in providing probiotic therapy recommendations.
Supp.AI	Dataset	Public	https://supp.ai/	[44]	AI-curated dataset of supplement-drug interactions automatically extracted from scientific literature.
SuppKG	Knowledge graph	Public	https://github.com/zhang-informatics/SemRep_DS/tree/main/SuppKG	[45]	A knowledge graph representing dietary supplement information with the purpose of discovering drug-supplement interactions.
TCDO (Traditional Chinese Drug Ontology)	Ontology	Public	https://bioportal.bioontology.org/ontologies/TCDO	[46]	Ontology with over 1500 terms, including terms drawn from existing ontologies and >1000 TCD-specific terms, labeled in both Chinese and English.
TCM-Mesh	Database	Public (unavailable)	http://mesh.tcm.microbioinformatics.org/	[47]	Database and analytical system designed for network pharmacology analysis of Traditional Chinese Medicine (TCM) preparations.
Traditional Chinese Medicine Integrative Database (TCMID)	Database	Public (unavailable)	http://www.megabionet.org/tcmid/	[48]	Provides information and bridges the gap between Traditional Chinese Medicine and modern life sciences.
Traditional Chinese Medicine Systems Pharmacology Database (TCMSP)	Database	Public	https://tcmsp-e.com/tcmsp.php	[49]	Database built based on the framework of systems pharmacology for herbal medicines, including 499 Chinese herbs.
Physical					
AcuFinder	Knowledge base	Public	https://www.acufinder.com/Acupuncture+Information	—	Knowledge base of articles on acupuncture, herbs & supplements, acupuncture points, etc.
AcuKG	Knowledge graph	Public	https://github.com/yimingli99/AcuKG-Knowledge-graph-for-medical-acupuncture	[50]	Knowledge graph that systematically organizes and represents acupuncture-related knowledge in a structured and scalable format.
ChiroIndex	Database	Public	https://chiroindex.org/#results	—	Specialized bibliographic database that indexes peer-reviewed literature produced by chiropractic publishers.
Complementary and Integrative Health Lexicon (CIHLex)	Dataset	Public	https://github.com/zhang-informatics/CIH	[51]	Lexicon developed to enhance the representation of physical and psychological CIH approaches in biomedical literature.
Psychological					
American Mindfulness Research Association (AMRA) Library and Mindfulness Research Monthly	Knowledge base	Membership web portal and monthly bulletin	https://goamra.org/library	—	Curated bibliography (> 5000 citations) and monthly bulletins summarizing new mindfulness-intervention studies; downloadable reference library for members.
Tai Chi Evidence Map	Knowledge base	Public (in manuscript)	No site	[52]	Evidence map that provides an overview of Tai Chi research and describes its volume and focus. It combines a systematic review of systematic reviews with a scoping review for the VA priority areas pain, posttraumatic stress disorder, and fall prevention.
Yoga for Pain and Mental Health	Knowledge base	Public (in manuscript)	No site	[53]	Evidence synthesis for yoga.

The resources are grouped by their focus area (comprehensive, nutritional, physical, and psychological) and within the focus area, sorted in alphabetical order.

Abbreviations: NCCIH, National Center for Complementary and Integrative Health; TCM, traditional Chinese medicine.

### Comprehensive resources

These resources focus broadly on CIH, rather than specific types of interventions.

*Global Wellness Institute*: is an evidence-based platform that evaluates the efficacy of wellness modalities like acupuncture, yoga, and meditation, and compiles studies from sources such as Cochrane Library [54]. It guides critical appraisal and supports informed use of complementary therapies.

*NatMed Pro*: provides healthcare professionals with detailed information on over 1400 natural ingredients and 250,000 commercial products, including data on safety, efficacy, interactions, and adverse effects. Interactive tools and multilingual handouts support clinical decision-making, with content regularly updated to reflect current research and regulations [55].

*The NCCIH A–Z Health Topics*: a public resource that catalogs over 100 health conditions and therapies, such as anxiety, osteoarthritis, acupuncture, and yoga. Designed for both clinical and public use, the index presents current evidence quality and supports the integration of complementary approaches through informatics [56].

*National Cancer Institute (NCI)* *Office of Cancer Complementary and Alternative Medicine (OCCAM*: an official NIH resource): The OCCAM, part of the NIH’s National Cancer Institute, supports evidence-based Complementary and Alternative Medicine (CAM) approaches in cancer prevention, treatment, and symptom management [57]. It provides peer-reviewed summaries and research updates on CIH therapies such as yoga and Traditional Chinese Medicine.

*VA Whole Health Library*: includes evidence maps and tables linking common conditions to effective integrative therapies. For example, low back pain is associated with yoga, acupuncture, and Tai Chi, whereas anxiety is linked to mindfulness, meditation, and yoga. Available through the Veteran's Administration (VA’s) Whole Health Library, these tools offer a quick-reference guide to evidence-based complementary treatments [58].

*WHO Traditional and Integrative Medicine Mapping*: a large-scale effort that mapped traditional, complementary, and integrative medicine (TCIM) interventions across various health conditions [59]. Published as a matrix dataset, these maps offer downloadable tables to help researchers and policymakers identify evidence-based TCIM approaches [60].

*The Lifestyle Factors Ontology (LFSO)*: the first comprehensive lifestyle medicine ontology organizes evidence-based, whole-person interventions for chronic disease prevention and treatment [17]. LFSO features a multihierarchical structure with detailed categories and integrates terms from resources like FoodOn [37], cross-referenced to over 50 biomedical ontologies. LFOS contains over 4000 lifestyle-related concepts and includes a dictionary-based Name Entity Recognition tool and can automate the extraction of lifestyle factors from biomedical literature [17,61].

### Nutritional resources

Nutritional interventions, such as dietary supplements and herbal therapies, have the strongest support in existing knowledge resources. We also include microbiome-related resources, given the active research linking diet to the microbiome’s key roles in metabolism, immunity, and mental health.

#### Diet-gut-microbiome resources

*curatedMetagenomicData*: this is an R/Bioconductor package, available via Bioconductor Experiment Hub, and offers curated standardized human microbiome data, including gene families, markers, pathways, and taxonomic profiles for analysis [18,62]. At the time of the publication of the original report [62], the dataset included samples from multiple body sites profiled by the Human Microbiome Project and from 25 other metagenomic studies, totaling 5716 samples that span 34 diseases and 28 countries.

*Diet-microbiome relation extraction*: a curated corpus developed to advance NLP research on diet-microbiome interactions, includes 165 scientific articles annotated with over 14,000 entities and 4200 relationships across 15 entity types (e.g., nutrients, microorganisms) and 13 relation types (e.g., increases, improves) [19].

*Disbiome*: a manually curated, open-access database developed at the University of Ghent, Disbiome maps microbial taxa to specific diseases based on published literature and catalogs microbiome–disease associations across 375 diseases and 1615 organisms from 10,866 experiments, organized by body site, abundance changes, and disease type [20,63].

*Food-gut microbiota disease interactions (FGMDI)*: a comprehensive database linking 313 foods, 1532 microbes, and 281 diseases through over 11,000 associations. Using graph attention networks, FGMDI predicts interactions and provides personalized dietary insights for microbiome-based health management [21].

*GMrepo*: this curated database includes over 71,000 annotated human gut metagenome samples from 353 projects, linked to 132 phenotypes. Select projects feature manually curated marker taxa, with the full dataset available as a downloadable tab-separated values (TSV) file [22,64].

*Gut microbiota watch*: a science communication knowledge base offering accessible evidence-based information on the gut microbiota’s role in health and disease [23]. Designed for healthcare professionals and the public, it highlights emerging research and global perspectives on digestion, immunity, and microbiome science.

*Gut microbial metabolite association with disease*: comprehensive database to identify and annotate the relationships among diseases, microbes, and metabolites to provide accurate and targeted solutions toward understanding the mechanism of complex disease and development of new markers and drugs [24].

*gutMGene*: a database that includes experimentally validated associations, both correlational and causal, among gut microbes, their metabolites, and host genes from the scientific literature [25]. The latest version of gutMGene, gutMGene v2.0, includes 1338 curated associations among 282 gut microbes, 278 microbial metabolites, and 238 host genes in humans [25].

*Human Gut Microbiome Atlas*: this program uses a systems biology approach to analyze oral and gut microbiomes across diverse cohorts, integrating metagenomic and multiomics data. By combining genomic, transcriptomic, proteomic, and metabolomic profiles, it aims to reveal host–microbiome interactions, identify disease-associated microbes, and support personalized interventions [65].

*Human Microbiome Compendium*: this dataset aggregates over 168,000 16S rRNA amplicon sequencing data for cross-study microbiome analysis and evaluation of geographic and technical effects on microbiome variation [26]. The 2025 version, includes data from 482 projects on 213 classes, 100 phyla, and 65 countries from 8 regions.

*Human Microbiome Project*: launched by the NIH in 2007, the Human Microbiome Project aimed to map microbial communities in the human body and their roles in health and disease [27]. In 2014, this initiative expanded this by generating multiomics data from disease-focused cohorts, sequencing 3000 bacterial genomes, and profiling over 300 individuals.

*Microbial Metabolites Database (MiMeDB)*: a freely available database containing detailed information about small molecule metabolites found in the human microbiome [28]. It contains metabolite, microbe, health, and bioactivity data and links to other databases, such as PubChem [66]. It contains >24K compounds, 1904 microbes, 626 diseases, and ∼6.6M genes.

*The Microsetta Initiative*: formerly the American Gut Project, The Microsetta Initiative, now based at UC San Diego, is a large citizen science effort mapping the human microbiome across global populations, using standardized protocols. It collected >15,000 self-reported samples from the United States, United Kingdom, and Australia, linking gut microbial diversity to lifestyle [67].

*Virtual Metabolic Human (VMH)*: this resource links genomic and metabolic information with disease pathways, enabling simulation of host-microbe interactions and dietary interventions [68]. VMH supports systems biology research and personalized health strategies through curated databases and computational modeling tools.

#### Food, dietary, and herbal supplement resources

*COlleCtion of Open Natural prodUcTs (COCONUT)*: launched in 2021, COCONUT 2.0 is an open-access platform for natural product data, featuring improved curation, a full redesign, and Findable, Accessible, Interoperable & Reusable (FAIR) compliance. It enables user submissions, community curation, and advanced searches, supporting research and discovery of bioactive compounds [31].

*Diet-Drug Interactions Database (DDID)*: a publicly accessible database cataloging 23,950 curated interactions between 1338 foods and herbs and 1516 drugs [32]. Each interaction is categorized by effect and sourced from over 3000 literature references, including Food and Drug Administration (FDA) labels and PubMed. This interface and links to DrugBank [69], PubChem [66], and UniProt [70]. DDID supports researchers and clinicians in identifying diet-drug interactions to improve safety.

*Encyclopedia of Traditional Chinese Medicine (ETCM)*: ETCM is a publicly accessible, structured database linking traditional Chinese medicine (TCM) with modern biomedical research. It includes data on 403 herbs, 3962 formulas, 7274 ingredients, 2266 target genes, and 3027 diseases, along with herb metadata. Mapping TCM ingredients to gene targets and pathways, ETCM supports systems (e.g., drug discovery) and aids in developing multitarget therapies aligned with TCM and precision medicine [33].

*FoodBall*: FoodBall is a European initiative using metabolomics to identify and validate biomarkers of food intake (BFIs) for improved dietary assessment. FoodBall also develops BFI validation criteria and offers informatics tools such as FooDB, PhytoHub, and Exposome-Explorer to enhance nutritional research, monitor dietary compliance, and support health claims [71].

*FoodData Central*: FoodData Central, released by the USDA in 2019, is a web-based resource offering 5 types of food and nutrient composition data. Designed for transparency and accessibility, it supports researchers, policymakers, health professionals, and product developers [35].

*FooDB*: developed by the Wishart Research Group, FooDB is an open-access database detailing over 1000 unprocessed foods and 28,000 food compounds and provides detailed data on nutrients, additives, flavor compounds, and health effects, including structure, properties, and food sources [71].

*FoodOn*: FoodOn is a global food ontology that standardizes food descriptions for consistent data retrieval and interoperability. Built on the FDA’s LanguaL system, it uses OWL-based formatting to integrate data across sources like USDA and EuroFIR, supporting international food research, regulation, and public health [37].

*High-throughput Experiment- and Reference-Guided Database*: this natural medicine database integrates high-throughput data with curated herb-target and herb–disease associations, supporting research on herbs’ effects on gene expression related to mental health, neuroinflammation, and stress. It offers searchable access to 7263 herbs, 49,258 ingredients, 12,933 genes, and 28,212 diseases, with tools for browsing, viewing, and downloading data [39].

*Indian Medicinal Plants, Phytochemistry And Therapeutics Database*: a curated database focused on Ayurvedic medicinal plants and their phytochemicals, compiled from over 100 traditional medicine texts and 7000+ research articles, includes 4010 plants, 17,967 phytochemicals, and 1095 therapeutic uses, with data mapped to specific plant parts [40].

*Integrated Dietary Supplements Knowledge Base:* A publicly available, standardized resource that consolidates data on dietary supplement ingredients, products, and their associations with drugs, diseases, symptoms, and therapeutic classes. The resource includes 4200 supplement ingredient concepts linked to 495 drugs, 776 diseases, and >137,000 products [41].

*Natural Product Drug Interactions (NaPDI)*: this database provides public access to scientific data, raw datasets, and recommended methods for evaluating the clinical significance of natural product-drug interactions and links NaPDI data across chemical characterization, metabolomics analyses, and pharmacokinetic studies (in vitro and clinical) [72].

*NIH Office of Dietary Supplements*: this platform provides evidence-based fact sheets on various vitamins, minerals, herbs, and other dietary supplements for both clinicians and the public [73]. Resources include the Dietary Supplement Label Database [74], which contains label information from thousands of United States products, and the PubMed Dietary Supplement Subset.

*Probiotics Reference Guide*: this guide was developed to support access to evidence-based information on commercially available probiotic products and provides evidence-based information on commercial probiotic products, integrating data from 556 clinical trials and 753 formulations, with 5708 links between products and studies [43].

*Supp*.*AI*: an AI-curated dataset that systematically extracts supplement-drug interactions from the scientific literature using NLP techniques encompasses 2044 dietary supplements, 2866 pharmaceutical drugs, and a total of 59,096 identified interactions to support pharmacovigilance, clinical decision support, and research on supplement-drug safety [44,75].

*SuppKG*: a curated knowledge graph representing comprehensive dietary supplement data to support the identification and analysis of drug-supplement interactions. SuppKG integrates information on ingredients, pharmacology, and interactions to enable computational reasoning, inform research, and enhance supplement safety [45].

*Traditional Chinese Drug Ontology*: this open-source, basic formal ontology aligned to support pharmacological research, safety assessment, and AI-driven discovery in Traditional Chinese Medicine. It includes over 1500 terms on decoction pieces, medicinal properties, and toxicity. Designed for semantic search, annotation, and knowledge mining, it powers TCM-semantic annotation system (SAS), which links literature to ontology terms for NLP and integration with biomedical data [76].

*TCM-Mesh*: provides information collected from various resources to enable network pharmacology for TCM preparations [47], including herbs, compounds, genes, and diseases, along with toxicity and gene–gene interaction data. The database seems comprehensive (6235 herbs, ∼380K compounds, 14,298 genes, and 6204 diseases); however, as of this writing, the web portal is no longer available.

*Traditional Chinese Medicine Integrative Database*: the database aggregates herb, ingredient, target, and disease data to enable network visualization of traditional prescriptions and biomedical connections [45]. It integrates data from various sources using text mining and links to drug and disease databases, including PubChem. The associated manuscript reports 8159 herbs, 25,120 compounds, 3791 diseases, and 6828 drugs, although the website was inaccessible at the time of writing.

*Traditional Chinese Medicine Systems Pharmacology Database*: this database captures relationships among TCM herbs, chemical ingredients, protein targets, and diseases, supporting pharmacokinetic modeling and herb–disease network construction [49,77]. It includes 499 herbs registered in the Chinese pharmacopoeia, with a total of 12,144 chemicals. For natural compounds, it includes information such as oral bioavailability, drug-likeness, intestinal epithelial permeability, and 3D structures.

### Physical resources

*AcuFinder*: a leading online resource for acupuncture and East Asian medicine, this platform offers detailed articles, research updates, and Frequently Asked Questions (FAQs) through its Learning Center. It covers techniques, herbal remedies, and condition-specific treatments for both practitioners and interested users [78].

*AcuKG*: AcuKG is a large-scale, structured knowledge graph of acupuncture, integrating over 1800 entities and 11,500 semantic relations from sources like WHO standards, PubMed, and AcuFinder. Using Optical Character Recognition (OCR), machine learning, ontology mapping, and LLM-assisted extraction, it links to SNOMED CT and MeSH to support evidence-based research and enhance AI tools for tasks like acupoint identification and domain-specific Q&A [50].

*ChiroIndex*: established in 1982, the ChiroIndex is a free database for chiropractic and manual therapy research. It offers advanced search features and access to full-text articles, supporting evidence-based practice for clinicians, educators, and researchers [79].

*Complementary and Integrative Health Lexicon*: a comprehensive, manually curated resource, encompassing 724 unique concepts and 885 terms (27% mapped to the Unified Medical Language System, was designed to enhance the representation of physical and psychological CIH modalities in biomedical literature [51].

### Psychological resources

Resources for psychological therapies like meditation and mind-body interventions are emerging, but remain limited in informatics databases. However, some network pharmacology tools indirectly support this research via neuroimmune pathways. Key sources are outlined below.

*American Mindfulness Research Association (AMRA)*: in 2019, the AMRA, published the *Mindfulness Research Monthly*, to summarize mindfulness studies current editions are accessible to AMRA members and subscribers [80].

*Tai Chi Evidence Map*: Solloway et al. [52] published an evidence map of Tai Chi research across health outcomes. This evidence was synthesized from 107 systematic reviews, highlighting strong support for Tai Chi in conditions like hypertension, cognitive function, osteoarthritis, depression, and chronic pain.

*Yoga for Pain and Mental Health*: evidence syntheses support yoga’s role in health. Duan-Porter et al. [53] mapped evidence on yoga for mental health, finding short-term benefits for depression but inconclusive results for anxiety and Post Traumatic Stress Disorder (PTSD).

## Discussion

We conducted a narrative review to identify knowledge resources that support CIH research, with particular emphasis on tools that generate or curate semistructured data suitable for AI- and informatics-driven analysis. Priority was given to resources that enable microbiome and multiomics integration and those applying informatics methods such as NLP and Knowledge Graphs (KGs). Because microbiome and omics datasets, as well as NLP- and KG-enabled resources, are inherently more structured, they are especially amenable to AI-driven precision nutrition and whole-person health application. Collectively, these findings highlight cross-domain informatics tools that advance evidence-based CIH and precision nutrition research while also identifying key opportunities and challenges for improving data interoperability, clinical translation, and equitable health outcomes.

### Opportunities

Open-source databases drive research and precision medicine by linking food, metabolites, and disease. Platforms such as FGMDI used together with MiMeDB may be beneficial for nutrition and microbiome research, as they provide free tools and high-quality datasets that researchers can use to cross-link data between food-metabolites-diseases and support translation to clinical practice. Accessibility to these resources (e.g., most are open-source) advances discoveries related to CIH approaches and the host-microbe interactions. Resources, such as MiMeDB, can support discovery and repurposing because metabolites produced by the microbiome play important roles in human health and disease. Future research should map how foods, probiotics, herbs, supplements, and drugs interact with microbiome-derived metabolites and influence disease outcomes [81]. Next, by creating a structured and interconnected knowledge base about diseases and the microbiome, these resources not only improve biomedical research but also enhance computational modeling and AI-driven predictions concerning microbiome and disease mechanisms. Resources (e.g., FGMCI, GMrepo, and MiMeDB) democratize metagenomic analysis by enabling researchers, without extensive computational resources, to process and interpret complex biological datasets [82]. By lowering technical barriers, the databases and resources can broaden participation in advanced scientific research, allowing a more diverse range of scientists to contribute to and benefit from ongoing advancements in the field.

### Challenges

Despite remarkable progress in CIH and microbiome research, several challenges limit the realization of its full potential. In the microbiome domain, first, establishing a universal definition of healthy gut flora remains difficult due to the inherent complexity of the gut microbiome [81,83]. This complexity is further compounded by varied diets, cultural practices, and environmental factors influencing microbiome composition. Integrating diverse datasets may help address this issue, but consistent data standards are necessary to maintain accuracy and compatibility across platforms. The current lack of standardized data across databases restricts the effective integration of multiple data sources, impeding scientific advancement [51]. Standardized data and using standards such as Web Ontology Language (OWL) and FAIR principles would enable data interoperability across all resources. Furthermore, many existing datasets underrepresent diverse global populations, limiting the generalizability of research findings [84]. Enhancing global participation by overcoming data collection barriers, forming partnerships with local communities, and adhering strictly to ethical sampling guidelines is essential. Fourth, most resources are manually curated from the literature (e.g., Disbiome). NLP can be an efficient solution for large data extraction and analysis of data from the biomedical literature, and support the curation of resources that would link CIH interventions (e.g., food, exercise, yoga, Tai Chi, etc.) to microbiome metabolites and diseases. In particular, for physical and psychological interventions, for which knowledge resources are very limited, NLP could serve to extend the existing limited evidence base [19,85]. Finally, translating CIH research into clinical practice remains a major challenge, highlighting the need to bridge this gap and ensure findings benefit healthcare practice [26,86]. Currently, in the CIH domain, there are limited structured computable resources to support an informatics approach of discovery and potential cross-reference with microbiome research [81].

### Future research and recommendations

As CIH and microbiome research continue to evolve, future efforts must focus on expanding population diversity, improving metadata quality, and developing interoperable data infrastructures. There is a critical need for enhanced informatics tools capable of integrating multiomic, clinical, and behavioral data across CIH domains. Emerging technologies, including AI, machine learning, knowledge graphs, and precision nutrition, offer powerful opportunities to synthesize complex data and uncover new therapeutic insights [[87], [88], [89]]. In parallel, greater emphasis on collaborative, interdisciplinary research and global data-sharing initiatives will be essential to ensure equitable innovation and translation into personalized, whole-person health solutions. For example, the integration of gut microbiome science with dietary research is reshaping the future of precision nutrition, offering the potential to tailor dietary interventions based on individual microbiome profiles to improve health outcomes [90]. Future work should prioritize the inclusion and use of standardized evaluation frameworks directed toward whole-person health to assess the efficacy, safety, and mechanisms of CIH therapies within diverse populations [91].

### Limitations

This review has several limitations. Although we identified and synthesized 47 key CIH interventions and microbiome resources, some, particularly proprietary or non-English language tools, may have been missed. As the field is rapidly evolving, this review offers a 2025 snapshot and may not reflect the latest updates or discontinuations. We did not assess the quality, usability, or interoperability of the data, which may limit our ability to evaluate how well they could be integrated into clinical, research, or informatics workflows. Future research should include structured appraisals of data quality metrics and interoperability frameworks to better inform researchers and practitioners regarding the strengths and limitations of each resource.

In conclusion, CIH research has reached a pivotal moment. Despite expanding resource networks, progress is hindered by limited diversity, inconsistent metadata, and fragmented data integration. Moving the field forward requires investment in inclusive datasets, standardized frameworks, and interoperable infrastructures. Emerging informatics approaches, such as NLP, KGs, and AI-driven predictive modeling, can accelerate discovery, support precision insights, and enable global collaboration. In particular, integrating CIH with advances in personalized nutrition and AI-powered analytics opens pathways to tailor interventions based on genetics, microbiome profiles, lifestyle, and consumer-generated health data. The resources reviewed highlight the urgent need to bridge complementary and conventional medicine, building toward next-generation, whole-person health solutions that deliver more personalized, equitable, and evidence-based outcomes.

## Author contributions

The authors’ responsibilities were as follows – RRA, MF: contributed to conceptualization, methodology, data curation, validation, formal analysis, investigation, writing – original draft, writing – review and editing; EG: contributed to data curation, validation, formal analysis, investigation, writing – original draft, writing – review and editing; RZ: contributed to funding acquisition, writing – review and editing; and HK: contributed to conceptualization, resources, supervision, project administration, funding acquisition, writing – original draft, writing – review and editing.

## Data availability

No new data were generated or analyzed in support of this research.

## Funding

This work was supported by funding from the National Center for Complementary and Integrative Health (NCCIH) and the Office of Data Science Strategy (ODSS) (grant number: U01AT012871). Its contents are solely the responsibility of the authors and do not necessarily represent the official views of the NCCIH, ODSS, and the NIH.

## Conflict of interest

The authors state that they have no competing interests to declare.

## References

[1] Herman P.M., Lavelle T.A., Sorbero M.E., Hurwitz E.L., Coulter I.D. (2019). Are nonpharmacologic interventions for chronic low back pain more cost effective than usual care? Proof of concept results from a Markov model. Spine.

[2] Langevin H.M., Weber W., Chen W. (2024). Integrated multicomponent interventions to support healthy aging of the whole person. Aging Cell.

[3] Langevin H.M. (2021). Moving the complementary and integrative health research field toward whole person health. J. Altern. Complement. Med..

[4] Stussman B.J., Black L.I., Barnes P.M., Clarke T.C., Nahin R.L. (2015). Wellness-related use of common complementary health approaches among adults: United States, 2012. Natl. Health Stat. Rep..

[5] Clarke T.C., Stussman B.J. (2018).

[6] Gannotta R., Malik S., Chan A.Y., Urgun K., Hsu F., Vadera S. (2018). Integrative medicine as a vital component of patient care. Cureus.

[7] Seifert G., Jeitler M., Stange R., Michalsen A., Cramer H., Brinkhaus B. (2020). The relevance of complementary and integrative medicine in the COVID-19 pandemic: a qualitative review of the literature. Front Med.

[8] Youn B.-Y., Song H.J., Yang K., Cheon C., Ko Y., Jang B.-H. (2021). Bibliometric analysis of integrative medicine studies from 2000 to 2019. Am. J. Chin. Med..

[9] National Center for Complementary and Integrative Health (2021).

[10] Fischer F.H., Lewith G., Witt C.M., Linde K., von Ammon K., Cardini F. (2014). High prevalence but limited evidence in complementary and alternative medicine: guidelines for future research. BMC Complement. Altern. Med..

[11] Greenhalgh T., Peacock R. (2005). Effectiveness and efficiency of search methods in systematic reviews of complex evidence: audit of primary sources. BMJ.

[12] An X., Bao Q., Di S., Zhao Y., Zhao S., Zhang H. (2019). The interaction between the gut microbiota and herbal medicines. Biomed. Pharmacother..

[13] Zhang B., Yue R., Chen Y., Yang M., Huang X., Shui J. (2019). Gut microbiota, a potential new target for Chinese herbal medicines in treating diabetes mellitus. Evid. Based Complement. Alternat. Med..

[14] Wei D., Xie L., Zhuang Z., Zhao N., Huang B., Tang Y. (2019). Gut microbiota: a new strategy to study the mechanism of electroacupuncture and moxibustion in treating ulcerative colitis. Evid. Based Complement. Alternat. Med..

[15] Sun Y., Ju P., Xue T., Ali U., Cui D., Chen J. (2023). Alteration of faecal microbiota balance related to long-term deep meditation. Gen. Psychiatr..

[16] Hoenders R., Ghelman R., Portella C., Simmons S., Locke A., Cramer H. (2024). A review of the WHO strategy on traditional, complementary, and integrative medicine from the perspective of academic consortia for integrative medicine and health. Front. Med..

[17] Nourani E., Koutrouli M., Xie Y., Vagiaki D., Pyysalo S., Nastou K. (2024). Lifestyle factors in the biomedical literature: an ontology and comprehensive resources for named entity recognition. Bioinformatics.

[18] Schiffer L. (2025). https://waldronlab.io/curatedMetagenomicData/.

[19] G. Hong, V. Hindle, N.M. Veasley, H.D. Holscher, H. Kilicoglu, DiMB-RE: mining the scientific literature for diet-microbiome associations. J Am Med Inform Assoc. 2025 Jun 1;32(6):998-1006. doi: 10.1093/jamia/ocaf054. PMID: 40152137; PMCID: PMC12089768. 10.1093/jamia/ocaf054 PMC12089768 40152137

[20] Janssens Y., Nielandt J., Bronselaer A., Debunne N., Verbeke F., Wynendaele E. (2018). Disbiome database: linking the microbiome to disease. BMC Microbiol.

[21] Zhang H., Zhang J., Zhao L., Bingqian Y., Hao Z., Wenwei L. (2024). Comprehensive database for food-gut microbiota-disease interactions (FGMDI) analysis and dietary recommendation applications. Food Biosci.

[22] D. Dai, GMrepo: a curated database of human gut metagenomes [Internet]. Available from https://gmrepo.humangut.info/home. (Accessed 23 March 2026).

[23] Gut Microbiota Watch, 2025 [Internet] https://www.gutmicrobiotawatch.org/gut-microbiota-info/. (Accessed 23 March 2026).

[24] Wang C.-Y., Kuang X., Wang Q.-Q., Zhang G.-Q., Cheng Z.-S., Deng Z.-X. (2023). GMMAD: a comprehensive database of human gut microbial metabolite associations with diseases. BMC Genom.

[25] Qi C., He G., Qian K., Guan S., Li Z., Liang S. (2025). gutMGene v2.0: an updated comprehensive database for target genes of gut microbes and microbial metabolites. Nucleic Acids Res..

[26] Lee S., Portlock T., Le Chatelier E., Garcia-Guevara F., Clasen F., Oñate F.P. (2024). Global compositional and functional states of the human gut microbiome in health and disease. Genome Res.

[27] Turnbaugh P.J., Ley R.E., Hamady M., Fraser-Liggett C.M., Knight R., Gordon J.I. (2007). The human microbiome project. Nature.

[28] The Microbial Metabolites Database (MiMeDB). MiMeDB. [Internet]. Available from: https://mimedb.org/ (accessed 23 March 2026).

[29] McDonald D., Hyde E., Debelius J.W., Morton J.T., Gonzalez A., Ackermann G. (2018). American Gut: an open platform for citizen science microbiome research. mSystems.

[30] Noronha A., Modamio J., Jarosz Y., Guerard E., Sompairac N., Preciat G. (2019). The virtual metabolic human database: integrating human and gut microbiome metabolism with nutrition and disease. Nucleic Acids Res.

[31] Chandrasekhar V., Rajan K., Kanakam S.R.S., Sharma N., Weißenborn V., Schaub J. (2025). COCONUT 2.0: a comprehensive overhaul and curation of the collection of open natural products database. Nucleic Acids Res.

[32] Hong Y., Xu H., Liu Y., Zhu S., Tian C., Chen G. (2024). DDID: a comprehensive resource for visualization and analysis of diet–drug interactions. Brief Bioinform.

[33] Xu H.-Y., Zhang Y.-Q., Liu Z.-M., Chen T., Lv C.-Y., Tang S.-H. (2019). ETCM: an encyclopaedia of traditional Chinese medicine. Nucleic Acids Res.

[34] Wishart D.S. (2024). Knowledge translation and knowledge mobilization from the FoodBAll project. Appl. Physiol. Nutr. Metab..

[35] US Department Agriculture (2025). https://fdc.nal.usda.gov/.

[36] Canadian Institutes for Health Research (2025). https://foodb.ca/.

[37] FoodOn (2025). https://foodon.org/.

[38] Danish Food Informatics. Langual Food Product Indexer. [Internet]. Available from: https://www.langual.org/langual_food_product_indexer_database_2017.asp (Accessed 15 April 2025).

[39] Bejing University of Chinese Medicine (2025). http://herb.ac.cn/.

[40] Institute of Mathematical Sciences (2021). https://cb.imsc.res.in/imppat/home.

[41] Rizvi R.F., Vasilakes J., Adam T.J., Melton G.B., Bishop J.R., Bian J. (2020). iDISK: the integrated DIetary supplements knowledge base. J. Am. Med. Inform. Assoc..

[42] Kellogg J.J., Paine M.F., McCune J.S., Oberlies N.H., Cech N.B. (2019). Selection and characterization of botanical natural products for research studies: a NaPDI center recommended approach. Nat. Prod. Rep..

[43] Goh A., Budijono B., Lim C., Chew E.H., Yap K. (2024). An in-house developed probiotics database E-reference information for healthcare professionals. Stud. Health Technol. Inform..

[44] L.L. Wang, O. Tafjord, A. Cohan, S. Jain, S. Skjonsberg, C. Schoenick, et al., SUPP.AI: Finding evidence for supplement-drug interactions, Proceedings of the 58th Annual Meeting of the Association for Computational Linguistics: System Demonstrations, 2020. Online. 362-371 :10.18653/v1/2020.acl-demos.41

[45] Schutte D., Vasilakes J., Bompelli A., Zhou Y., Fiszman M., Xu H. (2022). Discovering novel drug-supplement interactions using SuppKG generated from the biomedical literature. J. Biomed. Inform..

[46] Zhu J. (2021). TCDO: a community-based ontology for integrative representation and analysis of traditional Chinese drugs and their properties. Evid. Based Complement. Alternat. Med..

[47] Zhang R., Yu S., Bai H., Ning K. (2017). TCM-Mesh: the database and analytical system for network pharmacology analysis for TCM preparations. Sci. Rep..

[48] Xue R., Fang Z., Zhang M., Yi Z., Wen C., Shi T. (2012). TCMID: traditional Chinese medicine integrative database for herb molecular mechanism analysis. Nucleic Acids Res.

[49] Ru J., Li P., Wang J., Zhou W., Li B., Huang C. (2014). TCMSP: a database of systems pharmacology for drug discovery from herbal medicines. J. Cheminformatics.

[50] Li Y., Peng X., Peng S., Li J., Pei D., Zhang Q. (2026). AcuKG: a comprehensive knowledge graph for medical acupuncture. J. Am. Med. Inform. Assoc..

[51] Zhou H., Austin R., Lu S.-C., Silverman G.M., Zhou Y., Kilicoglu H. (2024). Complementary and integrative health information in the literature: its lexicon and named entity recognition. J. Am. Med. Inform. Assoc..

[52] Solloway M.R., Taylor S.L., Shekelle P.G., Miake-Lye I.M., Beroes J.M., Shanman R.M. (2016). An evidence map of the effect of Tai Chi on health outcomes. Syst. Rev..

[53] Duan-Porter W. (2016). Evidence map of yoga for depression, anxiety, and posttraumatic stress disorder. Hum. Kinet. J..

[54] Global Wellness Institute. Global Wellness Institute Wellness Evidence. [Internet]. Available from: https://globalwellnessinstitute.org/wellnessevidence/. (Accessed 23 March 2026)

[55] Therapeutic Research Center, Natural Medicines Database, Nat. Med. (2025) [Internet] https://naturalmedicines-therapeuticresearch-com.ezp1.lib.umn.edu/. (Accessed 23 March 2026).

[56] National Center Complementary and Integrative Health, Health Topics A-Z (2025) [Internet] https://www.nccih.nih.gov/health/atoz. (Accessed 5 March 2026).

[57] National Cancer Institute, Division of Cancer Treatment and Diagnosis. Office of Cancer Complementary and Alternative Medicine (OCCAM). [Internet]. Available from: https://cam.cancer.gov/ (Accessed 15 March 2026).

[58] Veterans Health Administration. Whole Health Library. [Internet]. Available from: https://www.va.gov/wholehealthlibrary/. (Accessed 23 March 2026).

[59] World Health Organization (WHO) (2019).

[60] Portella C.F.S., Ghelman R., Abdala C.V.M., Schveitzer M.C. (2020). Evidence map on the contributions of traditional, complementary and integrative medicines for health care in times of COVID-19. Integr. Med. Res..

[61] Whetzel P.L., Noy N.F., Shah N.H., Alexander P.R., Nyulas C., Tudorache T. (2011). BioPortal: enhanced functionality via new Web services from the National Center for Biomedical Ontology to access and use ontologies in software applications. Nucleic Acids Res.

[62] Pasolli E., Schiffer L., Manghi P., Renson A., Obenchain V., Truong D.T. (2017). Accessible, curated metagenomic data through ExperimentHub. Nat Methods.

[63] Disbiome. Link. Microbiome Dis. [Internet]. Available from: https://disbiome.ugent.be/home (Accessed 17 March 2026).

[64] A curated database of human gut metagenomes, GMrepo (2025) [Internet] https://gmrepo.humangut.info/home. (Accessed 17 March 2026).

[65] Human Gut Microbiome Atlas, 2025 [Internet] https://www.microbiomeatlas.org/. (Accessed 17 March 2026).

[66] PubChem, 2025 [Internet] https://pubchem.ncbi.nlm.nih.gov/. (Accessed 19 March 2026).

[67] University California San Diego (2025). https://microsetta.ucsd.edu/.

[68] Digital Metabolic Twins Centre. Virtual Metabolic Human. [Internet]. Available from: https://www.digitalmetabolictwin.org/copy-of-virtual-metabolic-human (Accessed 17 March 2026).

[69] DrugBank, 2025 [Internet] https://go.drugbank.com/. (Accessed 19 March 2026).

[70] UniProt. [Internet]. Available from: https://www.uniprot.org/ (Accessed 19 March 2026).

[71] Food Biomarker Alliance. FoodBall Portal. [Internet]. Available from: https://foodmetabolome.org/ (Accessed 15 March 2026).

[72] Natural Product-Drug Interaction Research. Center of Excellence for Natural Product-Drug Interaction Research (NaPDI). [Internet]. Available from: https://napdicenter.org/ (Accessed 15 March 2026).

[73] National Institute of Health, Office Dietary Supplements (2025). https://ods.od.nih.gov/.

[74] National Institute of Health, Office Dietary Supplements, Dietary Supplement Label Database (DSLD), 2025 [Internet] https://ods.od.nih.gov/Research/Dietary_Supplement_Label_Database.aspx. (Accessed 15 March 2026).

[75] Allen Institute for AI (2025). https://supp.ai/.

[76] (2021). Traditional Chinese Drug Ontology. Bioportal.

[77] Lab of Systems Pharmacology. TCMSP User Guide. Tradit. Chin. Med. Syst. Pharmacol. Database. [Internet]. Available from: https://www.tcmsp-e.com/load_intro.php?id=43 (Accessed 17 March 2026).

[78] AcuFinder. Acupunct. Referral Serv. [Internet]. Available from: https://www.acufinder.com/ (Accessed 17 March 2026).

[79] Index to Chiropractic Literature, ChiroIndex (2025) [Internet] https://chiroindex.org/#results. (Accessed 17 March 2026).

[80] American Mindfulness Research Association (2025). https://goamra.org/.

[81] Manske S. (2024). The microbiome: a foundation for integrative medicine, Integr. Med..

[82] Cooper K., Clarke M., Clayton J.B. (2023). Informatics for your gut: at the interface of nutrition, the microbiome, and technology. Yearb. Med. Inform..

[83] Ross F.C., Patangia D., Grimaud G., Lavelle A., Dempsey E.M., Ross R.P. (2024). The interplay between diet and the gut microbiome: implications for health and disease. Nat. Rev. Microbiol..

[84] Badal V.D., Wright D., Katsis Y., Kim H.-C., Swafford A.D., Knight R. (2019). Challenges in the construction of knowledge bases for human microbiome-disease associations. Microbiome.

[85] Diet-Microbiome Relation Extraction (DiMB-RE) (2025). Diet-Microbiome Relat. Extr. Sci. Lit..

[86] Aggarwal N., Kitano S., Puah G.R.Y., Kittelmann S., Hwang I.Y., Chang M.W. (2023). Microbiome and human health: current understanding, engineering, and enabling technologies. Chem. Rev..

[87] Saxena R., Sharma V., Saxena A.R., Patel A. (2024). Harnessing AI and gut microbiome research for precision health. J. Artif. Intell. Gen. Sci..

[88] K.L. Greathouse, A. Choudhury, Precision nutrition and the gut microbiome: harnessing AI to revolutionize cancer prevention and therapy, Cell Host Microbe. 33 (2925) 766–776. 10.1016/j.chom.2025.05.011 40505617

[89] Carlberg C., Blüthner A., Schoeman-Giziakis I., Oosting A., Cocolin L. (2025). Modulating biological aging with food-derived signals: a systems and precision nutrition perspective. Npj Aging.

[90] Sanz Y., Cryan J.F., Deschasaux-Tanguy M., Elinav E., Lambrecht R., Veiga P. (2025). The gut microbiome connects nutrition and human health. Nat. Rev. Gastroenterol. Hepatol..

[91] Ijaz N., Rioux J., Elder C., Weeks J. (2019). Whole systems research methods in health care: a scoping review, J. Altern. Complement. Med..

